# Boredom, art, and activism: notes on the experiences of people with disabilities in Mexico during the COVID-19 pandemic

**DOI:** 10.3389/fsoc.2023.1215106

**Published:** 2023-08-10

**Authors:** Beatriz Miranda-Galarza, Benjamín Mayer-Foulkes

**Affiliations:** Critical Disability Studies Programme, 17, Institute of Critical Studies, Mexico City, Mexico

**Keywords:** disability, boredom, Mexico, activism, artists

## Abstract

Confinement, imposed during the COVID-19 pandemic in Mexico, confronted most people with experiences of boredom. For people living with disability, however, the experience of boredom is not alien. Unfortunately, boredom and disability are topics frequently shrouded in taboo, constraining informative reflection and discussion. This perspective article briefly reflects on encounters and disagreements about these two categories in the lives of people with disabilities during the COVID-19 confinement in Mexico. The objective is to explore relevant elements for reflection and extend an invitation for critical and in-depth research on the matter.

## Introduction

Three years on from the COVID-19 pandemic and its collective experiences, research continues to explore understandings of everyday concepts, such as the individual, family and community, closeness and distance, illness and health, employment and unemployment, and life and death. These concepts need revisiting as, during the height of the pandemic, collective experiences reversed understandings and announced a radical civilizational transformation. However, this transformation has not materialized and reflections about life and habitation, particularly daily experiences, have either been obscured or are considered irrelevant. “Pandemic boredom” was a product of the involuntary isolation imposed in most countries that ushered in teleworking and tele-education. Despite alarming job losses and disruption of education, in countries like Mexico (Sánchez-Talanquer et al., 2021), the phenomenon of boredom has not been explored, especially among marginalized populations, including people living with a disability (Meresman and Ullmann, 2020). Not critically exploring this “pandemic-boredom-disability” relationship prevented realities, experienced by this population, being made public and inhibited the implementation of timely actions. Furthermore, the opportunity to learn from strategies employed by these people, who constantly face isolation and boredom imposed by society and states, was lost. This article exposes elements needing consideration when developing research about boredom in their lives and highlights the knowledge contribution these people can make to society in general.

## Boredom and disability: brief approach

The juncture between disability and boredom refers to a complex structure, impossible to locate in a single area of study or reflection. These two concepts, namely disability and boredom, are marked by the authority of historical discourses fluctuating between moralizing-medicalizing and the political-ethical, that consider the subjectivity of a desiring subject and the relation to its context. Kessel (2001) argues boredom as a modern concept originated in the 18th century in the form of tedium or hopelessness but has been envisioned since the Middle Ages, a period in which “doing nothing” was positive in the case of upper-class men or artists, but a sin in women. The two world wars and expansion of technological capitalism has witnessed boredom circulate as a synonym of social illness, pathologizing the individual (Ros Velasco, 2022). Historically, however, in relation to disability, it has been defined by religious-moral and medical-psychiatric frameworks positioning it as divine or sinful, framing it as disease and abnormality. Proponents of the social model of disability (Oliver, 1999) could argue categorizing it this way aligns with the 18th century Western perceptions that resulted in the expulsion of “non-productive” and “useless” bodies from production processes during the First Industrial Revolution. Enlightenment institutionalized medical discourse and prevented individuals, considered to be abnormal, from actively participating in public and political life (Davis, 1995). Medical and psychiatric institutions took charge of these lives, and their destinies oscillated between being normalized and institutionalized (Snyder and Mitchell, 2019).

Classic contributions from psychology refer to boredom as an emotion arising from the loss of the ability to be amazed (Antón, 2012) and has become a synonym for depression, anguish, or laziness. Schopenhauer postulated boredom is more closely linked to the human will and dependent on their resolution not their needs (Schopenhauer, 2006). Schopenhauer claimed humans have two potential responses to the problems life presents: one, leave them unresolved and suffer, or two, solve them and live with boredom until a new problem replaces the resolved one. Thus, according to the philosopher, life exists between suffering and boredom. From this rather pessimistic position, Retana (2011) recovers the political and ethical texture of boredom. For the author, the constant search to fill the gap that satisfaction of a need leaves is mediated not only by individual will and social interactions, as Schopenhauer argued, but also by affective, political, economic, and cultural ones. From this point of view, power games and moral and ethical control also contribute to determining boredom. Thus, Retana maintains boredom can be placed in a group of “forbidden emotions” (Retana, 2011, p. 185) that “should” have no place in human life. Faced with this contribution of structural elements, psychoanalytic perspectives define boredom more broadly. For Lacan (2011), boredom is sustained by desire, “the desire for something else” (Antón, 2012), and this desire can be conceived as a threshold for life itself. Antón, expanding on Lacan's position, asserts boredom “makes it possible to take a position, although it does not guarantee it and it can lead us to an entrance door to establish a social bond” (Antón, 2012, p. 105).

But what happens when the subject is considered “non-desiring,” as is the case for people with disabilities? Would it be inferred that he or she is incapable of taking a position toward life and therefore incapable of building social bonds? Should one think that the person lives in a permanent state of non-awareness in the face of his or her own boredom? A legacy of the Enlightenment was to portray the disabled person as devoid of rationality, intention, and desire, in other words, of subjectivity. Wilton (2003), assuming a psychoanalytic approach to physical disability, maintains disability in general is posited as a “symbolic substitute” for castration, in the Freudian and Lacanian sense, and therefore a disabled body is always imagined as “a-desiring.” From this perspective, boredom would then be considered as either intrinsic to the character of the disabled person as it was considered for women in the Middle Ages, or non-existent, in terms of the absence of its human conformation.

## Boredom and disability during the pandemic in Mexico

In Mexico, a 2020 report from the Social Sciences Observatory of the Mexican Council of Social Sciences indicated the measures taken during the pandemic by the government and private organizations were aimed at “trying to reduce the duration of social isolation to avoid the appearance of emotions such as boredom and anger, as well as increases in family conflicts or intrafamily violence” (Santillán Torres, 2021). At the same time, Mexican society, like many others in the world, was subject to persistent digital stimuli, a phenomenon coined as “pandemic boredom” by many media outlets such as the New York Times, El Mundo, and El País. Isolation, the extreme limitation of social contact, the reduction of economic and relational activities, and restriction of access to health services resulted for many in an overwhelming “new normal.” However, for most people with disabilities, especially institutionalized people, this was just another aspect of their all too familiar “normality” (Sharma and Rau Barriga, 2021). Approximately 20 million disabled people live in Mexico, comprising 16.5% of its population. Of this, 53% are women, 47% are men (Plataforma Dis-capacidad, 2021), and 50% live in poverty. According to the “Parallel Victims: Those Affected Who Are Not Spoken” project, the pandemic exacerbated the isolation conditions of people with disabilities, limited their access to medical and rehabilitation services, their necessities for on-line education programs established by the government were overlooked. For many people with disabilities, rehabilitation, education, and health service spaces constitute the only places open for socializing and establishing relational bonds. Staying at home for longer than usual exacerbates feelings of frustration and boredom, sometimes manifesting in aggressive attitudes resulting from disruption of daily dynamics (Solis Garcia, 2022). It intensified the situation where risks were exposed due to lack of accessible information about the pandemic for people with disabilities and their families. An illustration of such risks include the non-existence of transparent masks for deaf people which exacerbated misinformation and isolation. Experiences of tedium, depression, and boredom resulted in the increased administration of psychotropic medications used for depression and anxiety (Solis Garcia, 2022, p. 13). The thematic report “*Infancias Encerradas* (Forgotten childhoods),” conducted by the Human Rights Commission of Mexico City (CNDH, 2020) involving more than 1,300 children and adolescents, revealed four out of 10 played video games, three out of 10 chatted with their friends, two out of 10 talked on the phone, and two out of 10 read for pleasure. Moreover, the findings indicated only four out of 10 of the participants were happy with these activities during their period of confinement, and people with multiple disabilities were the least involved in activities that generated happiness. Finally, it was reported that children with disabilities were less happy in confinement than those without disabilities. Despite these findings, the effects of confinement and the pandemic on people with disabilities were obscure and much less understood by society in general and the government (Artigues, 2021).

## Artistic creation and activism in response to desire

In unexpected ways in Mexico, among people with disabilities, boredom during the pandemic affirmed the psychoanalytically posed constant desire for “something else.” Isolation, the constant risk of dying, not only in relation to the pandemic but because of limited access to basic services, and the exacerbation of boredom reported by many people with disabilities due to the limitations imposed on them provoked two unique responses: artistic and activist. Artigues (2021) reports how activism took on a new meaning for people with disabilities who were contending with total abandonment by the government during the pandemic. The following examples illustrate this: On March 17, 2020, the deaf community filed a legal protection order before the Thirteenth District Court for Administrative Matters of Mexico City demanding access in sign language to information about the pandemic and the country's situation. This action forced the government to open a microsite accessible to people with disabilities in general. Likewise, another protest carried out by various organizations of people with disabilities managed to get the government to implement actions for school-age populations with disabilities and created a website where disabled and indigenous populations could access educational material relevant for their needs. Another field of activism by people with disabilities related to access to vaccines; this led to the provision of vaccine information, the prioritization of people with disabilities, the assurance of physical accessibility to vaccination sites, and the possibility for being vaccinated at home.

Additionally, groups of young women and disabled university students focused on action about social networks. An article in the newspaper El País in 2020 regarding online activism during confinement stated platforms like Change.Org (Prada, 2020) reported an exponential increase in the activity of social organizations or individual activists during the first quarter of confinement. Other platforms such as Osoigo.Org reported an increase in demands for support for people with disabilities or living with illness (Prada, 2020). In Mexico, the emergence of initiatives such as Rangel and Blanco (2022), the National Network of Feminists with Disabilities, occurred alongside the formation of *No Es Igual*, a collective organized by artists from different artistic disciplines, regions of the country, and people with disabilities. The emergence of these initiatives, according to some of its members, occurred when people with disabilities were encouraged to defend their rights. Furthermore, the desire to survive mentally and materially during the pandemic (17, Institute of Critical Studies, 2020) stimulated this action. Seminars, online talks, manifestos, and collective and individual artistic projects circulated on social networks, alerting the public to the precarious circumstances in which most people with disabilities lived through the pandemic. This type of response to boredom also facilitated connections to other groups with similar objectives in other Latin American countries.

The pandemic-induced boredom encountered by individuals with disabilities in Mexico has exposed the prevailing taboos surrounding their lives, subjectivity, and everyday existence. It has also revealed the inertia on the part of the government and society to critically examine these issues. The then United Nations Rapporteur on the rights of persons with disabilities, Catalina Devandas, denounced the exclusion of people with disabilities from governmental pandemic strategies, emphasizing their feeling of being “left behind.” In response to this abandonment, the disabled community has embraced imposed boredom as a catalyst to unite, take action, and engage in activism and artistic endeavors. Consequently, boredom, as a reaction to the yearning for something more—such as emancipation—has opened a pathway for an ongoing process that will undoubtedly yield results in the future. However, information regarding this topic is scarce, underscoring the importance of conducting critical research on the link between boredom and disability. Such research can inform work programs and facilitate learning from the lived experiences of individuals with disabilities.

## Discussion

In the face of discourse that overlooks boredom as a phenomenon experienced by individuals with disabilities, recent voices acknowledge the disabling circumstances created by political, economic, cultural, and social barriers that affect people with physical, sensory, or intellectual impairments. The pandemic-induced boredom experienced by people with disabilities in Mexico exposed taboos permeating their lives, subjectivity, and daily existence. It has also exposed the government's inertia and the inability of society to critically examine these issues. Catalina Devandas, at that time the United Nations Rapporteur on the rights of persons with disabilities, denounced the exclusion of people with disabilities from government strategies about dealing with the pandemic and emphasized how people with disabilities felt that they were “left behind” (Lizama, 2020). Responding to this abandonment, the disabled community embraced the imposed boredom, using it as a catalyst to unite, engage in activism and generate artistic creations. Consequently, boredom as a reaction to the emptiness generated by the desire for something more, for example emancipation, opened the door to an ongoing process that will undoubtedly lead to positive social change in the future. Information about it, however, is scarce and underscores the need for critical research on the link between boredom and disability. Such research can inform various programmes and facilitate learning from lived experiences of people with disabilities.

## Data availability statement

The original contributions presented in the study are included in the article/supplementary material, further inquiries can be directed to the corresponding authors.

## Author contributions

All authors listed have made a substantial, direct, and intellectual contribution to the work and approved it for publication.
